# ABA Renewal Involves Enhancements in Both GluA2-Lacking AMPA Receptor Activity and GluA1 Phosphorylation in the Lateral Amygdala

**DOI:** 10.1371/journal.pone.0100108

**Published:** 2014-06-12

**Authors:** Kyungjoon Park, Beomjong Song, Jeongyeon Kim, Ingie Hong, Sangho Song, Junuk Lee, Sungmo Park, Jihye Kim, Bobae An, Hyun Woo Lee, Seungbok Lee, Hyun Kim, Justin C. Lee, Sukwon Lee, Sukwoo Choi

**Affiliations:** 1 School of Biological Sciences, College of Natural Sciences, Seoul National University, Seoul, Korea (ROK); 2 Center for Neural Science and Center for Connectomics, Korea Institute of Science and Technology, Seoul, Korea (ROK); 3 Department of Anatomy and Brain Korea 21 Biomedical Science program, Korea University, College of Medicine, Seoul, Korea (ROK); 4 Department of Cell and Developmental Biology, Dental Research Institute, Seoul National University, Seoul, Korea (ROK); Radboud University, Netherlands

## Abstract

Fear renewal, the context-specific relapse of fear following fear extinction, is a leading animal model of post-traumatic stress disorders (PTSD) and fear-related disorders. Although fear extinction can diminish fear responses, this effect is restricted to the context where the extinction is carried out, and the extinguished fear strongly relapses when assessed in the original acquisition context (ABA renewal) or in a context distinct from the conditioning and extinction contexts (ABC renewal). We have previously identified Ser831 phosphorylation of GluA1 subunit in the lateral amygdala (LA) as a key molecular mechanism for ABC renewal. However, molecular mechanisms underlying ABA renewal remain to be elucidated. Here, we found that both the excitatory synaptic efficacy and GluA2-lacking AMPAR activity at thalamic input synapses onto the LA (T-LA synapses) were enhanced upon ABA renewal. GluA2-lacking AMPAR activity was also increased during low-threshold potentiation, a potential cellular substrate of renewal, at T-LA synapses. The microinjection of 1-naphtylacetyl-spermine (NASPM), a selective blocker of GluA2-lacking AMPARs, into the LA attenuated ABA renewal, suggesting a critical role of GluA2-lacking AMPARs in ABA renewal. We also found that Ser831 phosphorylation of GluA1 in the LA was increased upon ABA renewal. We developed a short peptide mimicking the Ser831-containing C-tail region of GluA1, which can be phosphorylated upon renewal (GluA1_S_); thus, the phosphorylated GluA1_S_ may compete with Ser831-phosphorylated GluA1. This GluA1_S_ peptide blocked the low-threshold potentiation when dialyzed into a recorded neuron. The microinjection of a cell-permeable form of GluA1_S_ peptide into the LA attenuated ABA renewal. In support of the GluA1_S_ experiments, a GluA1_D_ peptide (in which the serine at 831 is replaced with a phosphomimetic amino acid, aspartate) attenuated ABA renewal when microinjected into the LA. These findings suggest that enhancements in both the GluA2-lacking AMPAR activity and GluA1 phosphorylation at Ser831 are required for ABA renewal.

## Introduction

Fear-related emotional disorders, such as PTSD and phobia, are clinically challenging to treat because the symptoms strongly relapse even after extensive exposure-based therapy [1], [2]. Fear renewal is one of the most promising animal models of fear relapse, wherein pre-acquired fear is attenuated by extinction but later relapses without explicit relearning [3]. Together with other animal models, such as reinstatement and spontaneous recovery, renewal has been widely investigated at the systems and behavioral levels [4]–[7]. To avoid contextual influences, extinction is often carried out in a different context from the original fear conditioning. The extinguished fear can relapse when the subject is presented with a conditioned stimulus (CS) in the same context in which the fear conditioning was performed (ABA renewal) or in a third context distinct from the context where the fear conditioning or extinction was carried out (ABC renewal). Although both ABA and ABC renewal demonstrate the context-dependency of extinction learning, their mechanisms and manifestations have been shown to differ clearly in several aspects [8]–[14]. The dorsal hippocampus plays a critical role in ABC renewal [15], [16], but not ABA renewal [4], [17]. In addition, blockade of kappa opioid receptor in the ventral hippocampus has a significant effect on ABA renewal, but not ABC renewal [7], [8]. Thus, it is important to study these two forms of fear renewal independently. Clinically, ABA renewal can be particularly important because it is well defined in humans [11], and PTSD patients often experience flashbacks that are triggered by exposure to the contextual aspects of traumatic memories [18].

The LA is known to be an important brain structure where CSs and unconditioned stimuli are associated during the acquisition of fear memory [19]. Lesions or inactivation of the LA result in attenuation in fear conditioning [20], [21]. The thalamic input synapses onto the lateral amygdala (T-LA synapses); the T-LA synapse is known to transmit acoustic CS information to the whole amygdaloid complex, is potentiated upon fear learning [22], [23], and is depotentiated by fear extinction [24], [25] in concert with a change in the neural network between the basolateral amygdala, the ventral hippocampus, and the prefrontal cortex [5], [6], [26]–[28]. Although the mechanisms underlying fear acquisition and extinction have been well defined, the synaptic and molecular mechanisms underlying fear renewal remain relatively unknown.

In our recent study on ABC renewal [29], we have shown that Ser831 phosphorylation of GluA1 in the LA is required for renewal and that the activity of GluA2-lacking AMPARs is enhanced upon renewal. Since ABA renewal has been shown to differ from ABC renewal in several aspects, it is critical to determine whether Ser831 phosphorylation of GluA1 in the LA is also required for ABA renewal. It also remains to be elucidated whether the activity of GluA2-lacking AMPARs is required for renewal. Here, we have examined the molecular and synaptic mechanisms underlying ABA renewal. Our results provide strong evidence that the activity of GluA2-lacking AMPARs is enhanced during and required for ABA renewal. Similarly to ABC renewal, ABA renewal involves enhancements in the Ser831 phosphorylation of GluA1 in the LA.

## Results

### Enhancement of T-LA Synaptic Efficacy upon ABA Renewal

**Figure 1:**
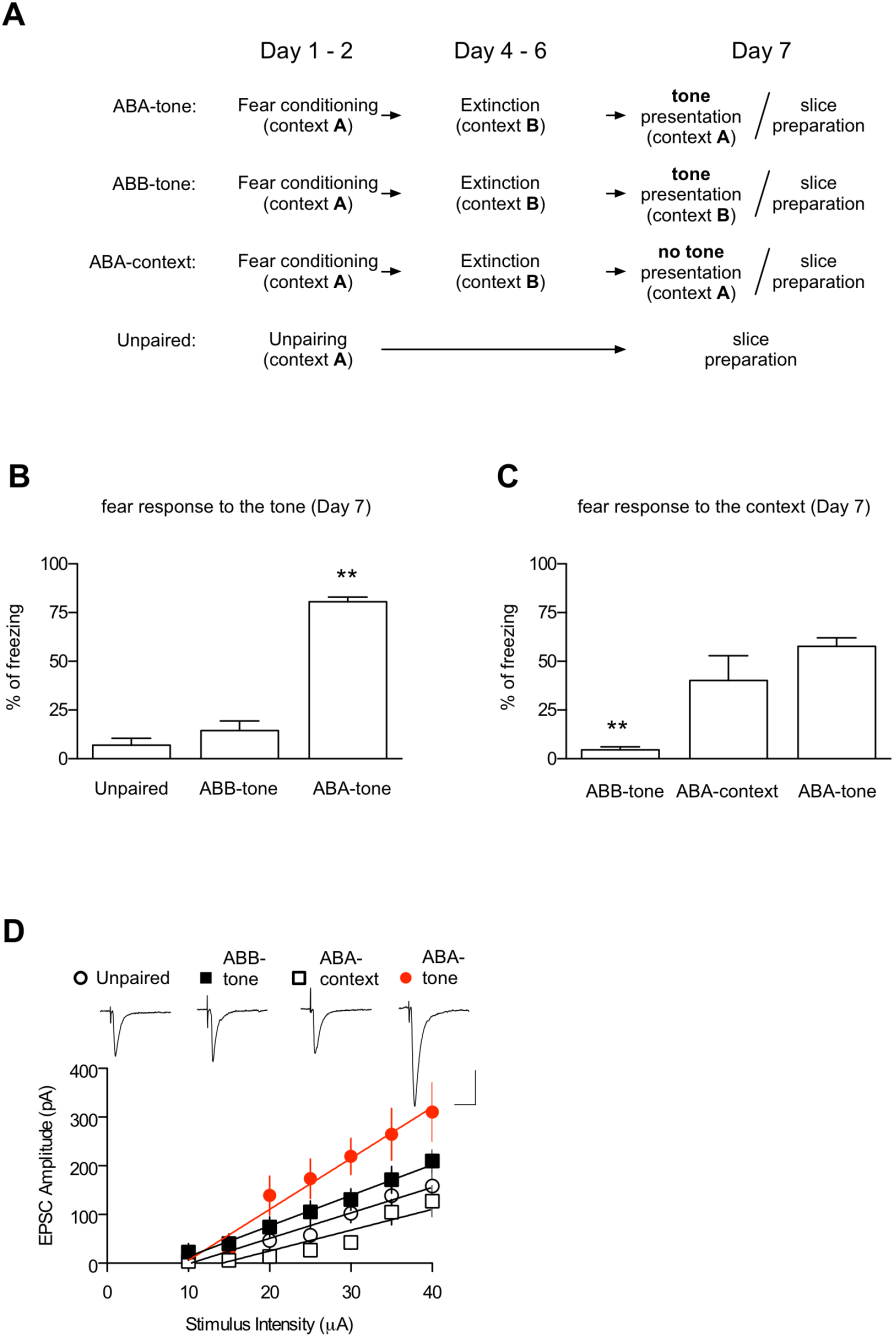
ABA renewal-inducing stimuli produce a context-dependent enhancement of synaptic efficacy at T-LA synapses. **A.** The behavioral procedure. On Day 7, brain slices were prepared immediately after a tone test (ABB-tone and ABA-tone group) or context exposure (ABC-context group). In unpaired controls, one set of rats was killed for the preparation of brain slices, while another set was monitored for conditioned freezing to a CS on Day 7. **B** and **C.** Pooled behavioral results. **, p<0.01. **D.** Input-output curves for EPSCs in unpaired controls (n = 7), ABC-context (n = 7), ABB-tone (n = 16) and ABA-tone groups (n = 9). The representative traces are the averages of four responses evoked by input stimulations of 35 µA. Scale bars, 20 ms and 100 pA.

We first determined whether renewal is associated with synaptic efficacy changes at the T-LA synapses, a major input synapse onto the whole amygdaloid complex. Different controls (tone controls, context controls, unpaired controls) were used to ensure that the effects observed with the renewal protocol required both the tone presentation and conditioning context (Figure 1A). Tone controls (ABB-tone) differed from the renewal group (ABA-tone) in that rats were acclimated (10 min) and tone-tested in the extinction context rather than in the conditioning context. Context controls (ABA-context) involved the same protocol as the renewal group, except that rats were not tone-tested. Cued or contextual fear was assessed (Figure 1B, C). Synaptic efficacy was assessed using whole-cell voltage-clamp recordings in the slices prepared immediately after a tone test or context exposure. The renewal protocol, which successfully induced a strong relapse of extinguished fear, produced a significant potentiation at the T-LA synapses compared with the other three controls (F_3,35_ = 4.357, p = 0.0104, one-way ANOVA; unpaired, 5.23±0.83 pA/µA; ABB-tone, 6.28±0.77 pA/µA; ABA-context, 4.27±1.06 pA/µA; ABA-tone, 10.47±1.99 pA/µA; p<0.05 for all the three pairs, Newman-Keuls post-test; Figure 1C); there was no significant difference between the three controls (p>0.05 for all designated pairs, Newman-Keuls posttest). We also compared the series resistances of the whole-cell recordings (unpaired, 15.46±1.73 MΩ; ABB-tone, 15.74±0.89 MΩ; ABA-context, 14.19±1.32 MΩ; ABA-tone, 15.19±0.76 MΩ) between the four groups, and they were not significantly different (F_3,35_ = 0.33, p = 0.80, one-way ANOVA). Taken together, the strengthened synaptic efficacy on the T-LA pathway appears to be firmly associated with ABA renewal.

**Figure 2:**
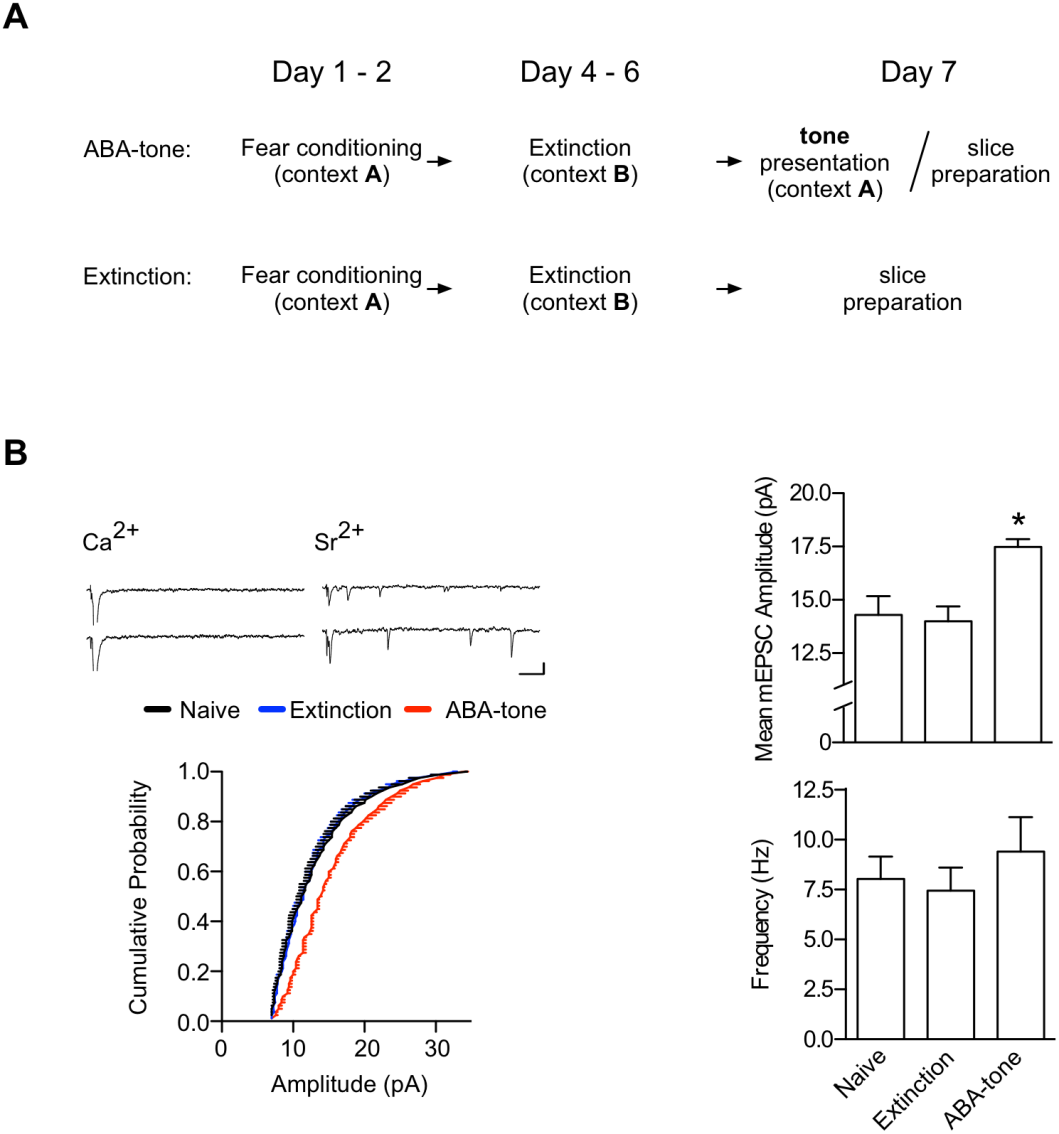
ABA renewal-inducing stimuli enhance the amplitude of AMPAR-mediated mEPSCs at T-LA synapses. **A.** The behavioral procedure. On Day 7, brain slices were prepared immediately after a tone test (ABA-tone group). In the extinction group, one set of rats was killed for the preparation of brain slices, while another set was monitored for conditioned freezing to CS on Day 7. **B.** Upper left: Sample traces of evoked EPSCs in the presence of Ca^2+^ or Sr^2+^. Scale bars, 50****ms and 10 pA. Note that the representative traces were further processed with a digital Gaussian filter for better display. Lower left: Cumulative amplitude distributions of evoked mEPSCs in the presence of Sr^2+^ (n = 300 events per cell). Upper right: Mean amplitude of mEPSCs evoked in the presence of Sr^2+^ (F_2,16_ = 5.896, p = 0.0121; naïve, 14.3±0.9 pA, n = 7; extinction,14.0±0.7 pA, n = 7; ABA-tone, 17.5±0.4 pA, n = 5; *, p<0.05). Lower right: Mean frequency of mEPSCs evoked in the presence of Sr^2+^ (naïve, 8.0±1.1****Hz, n = 7; extinction, 7.4±1.2****Hz, n = 7; ABA-tone, 9.4±1.7****Hz, n = 5).

To investigate whether the observed enhancement of T-LA synaptic efficacy is due to a presynaptic or postsynaptic change before and after ABA renewal, we examined miniature AMPAR-mediated EPSCs (mEPSCs) that were sampled from evoked T-LA synapses in three groups: naïve, extinction, and ABA-tone (renewal) (Figure 2A). Although no significant difference in the frequency of asynchronous mEPSCs was observed between the three groups (F_2,16_ = 0.5348, p = 0.5959, one-way ANOVA; p>0.05 for all the pairs designated, Newman-Keuls post-test), the mean amplitude distribution of mEPSCs was significantly shifted to the right in the renewal group relative to either naïve controls or the extinction group (p<0.001 for the naïve-renewal pair and the extinction-renewal pair, p>0.2 for the naïve-extinction pair, Kolmogorov-Smirnov test; Figure 2B). These results indicate that a postsynaptic function (i.e., AMPA receptor function and/or number) may be enhanced upon renewal.

### Enhancements in the Activity of GluA2-lacking AMPARs during ABA Renewal or its Cellular Substrate, Low-threshold Potentiation

**Figure 3:**
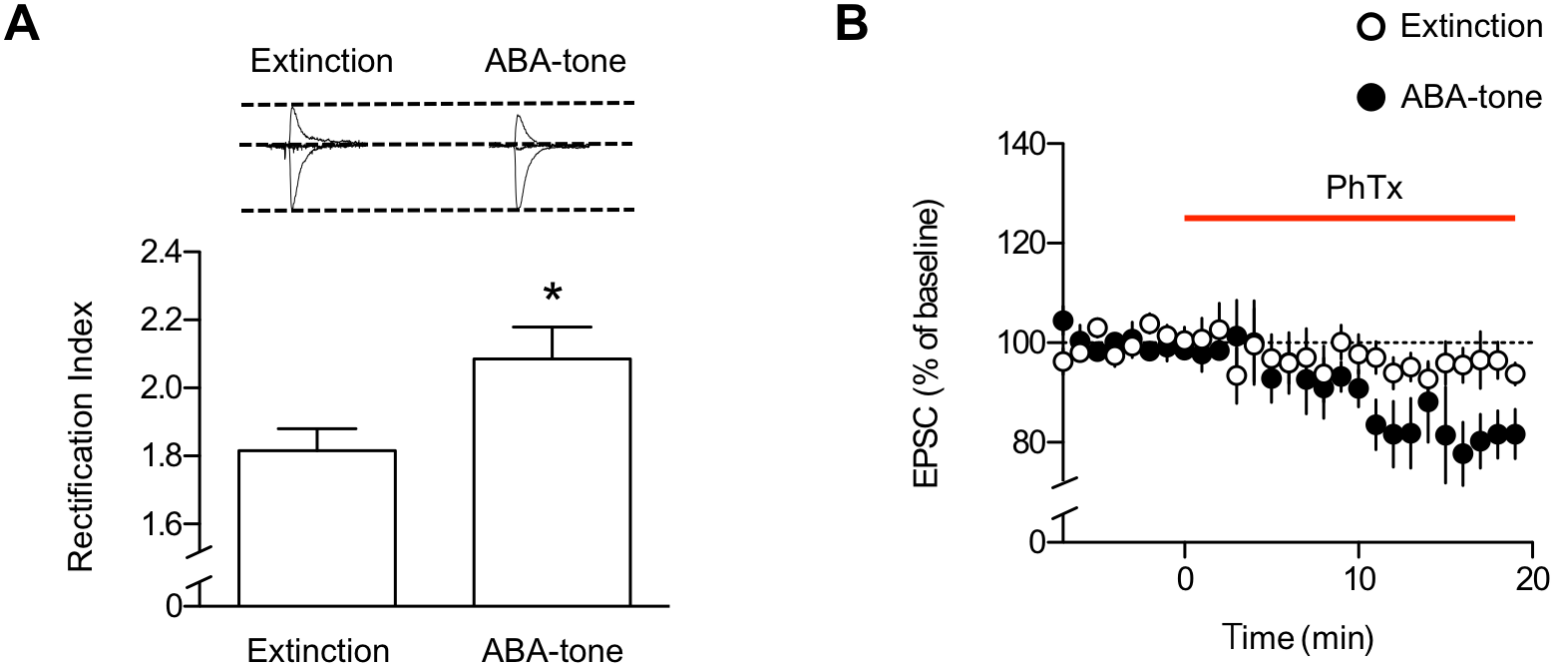
ABA renewal-inducing stimuli enhance GluR2-lacking AMPAR activity at T-LA synapses. **A.** ABA renewal-inducing stimuli increased the RI of synaptic AMPA currents compared with the extinction group (extinction, n = 13; ABA-tone, n = 12). Superimposed averages of AMPAR-mediated EPSCs recorded at −60 mV and +40 mV are shown in the inset (see Methods for additional details). **B.** PhTx, a selective blocker for GluA2-lacking AMPARs, inhibited AMPAR-mediated EPSCs in the renewal group, but not in the extinction group. D-AP5 (100 µM) was included in the recording solution. EPSCs were elicited at a frequency of 0.33 Hz. *, p<0.05 (paired t-test).

To examine changes in the subunit composition of AMPA receptors following ABA renewal, we examined the rectification index of AMPAR-mediated EPSCs. This method is based on the fact that AMPARs lacking GluA2 subunits display inward rectification due to the voltage-dependent block of the channel pore by polyamines at positive membrane potentials [30]–[33]. We compared the rectification index between the extinction and renewal groups using a spermine-containing intracellular solution. EPSCs at the LA synapses in rat brain slices prepared after extinction showed an RI of 1.82±0.06, whereas EPSCs in slices prepared immediately after fear renewal (ABA-tone group) exhibited a significantly higher RI of 2.09±0.09 (p<0.05, unpaired t-test, Figure 3A) without significantly changing the reversal potentials (14.77±0.78 mV, n = 13, extinction; 14.83±0.95 mV, n = 12, renewal; p>0.9, unpaired t-test). Moreover, the application of 10 µM philanthotoxin 433 (PhTx; a selective blocker for GluA2-lacking AMPARs [34]) inhibited AMPAR-mediated EPSCs to a substantially greater degree in the ABA-tone group (n = 4 from 3 rats) compared with the extinction group (n = 4 from 3 rats, p<0.05, unpaired t-test; Figure 3B). These results suggest that the activity of GluA2-lacking AMPARs at the LA synapses was enhanced upon ABA renewal.

**Figure 4:**
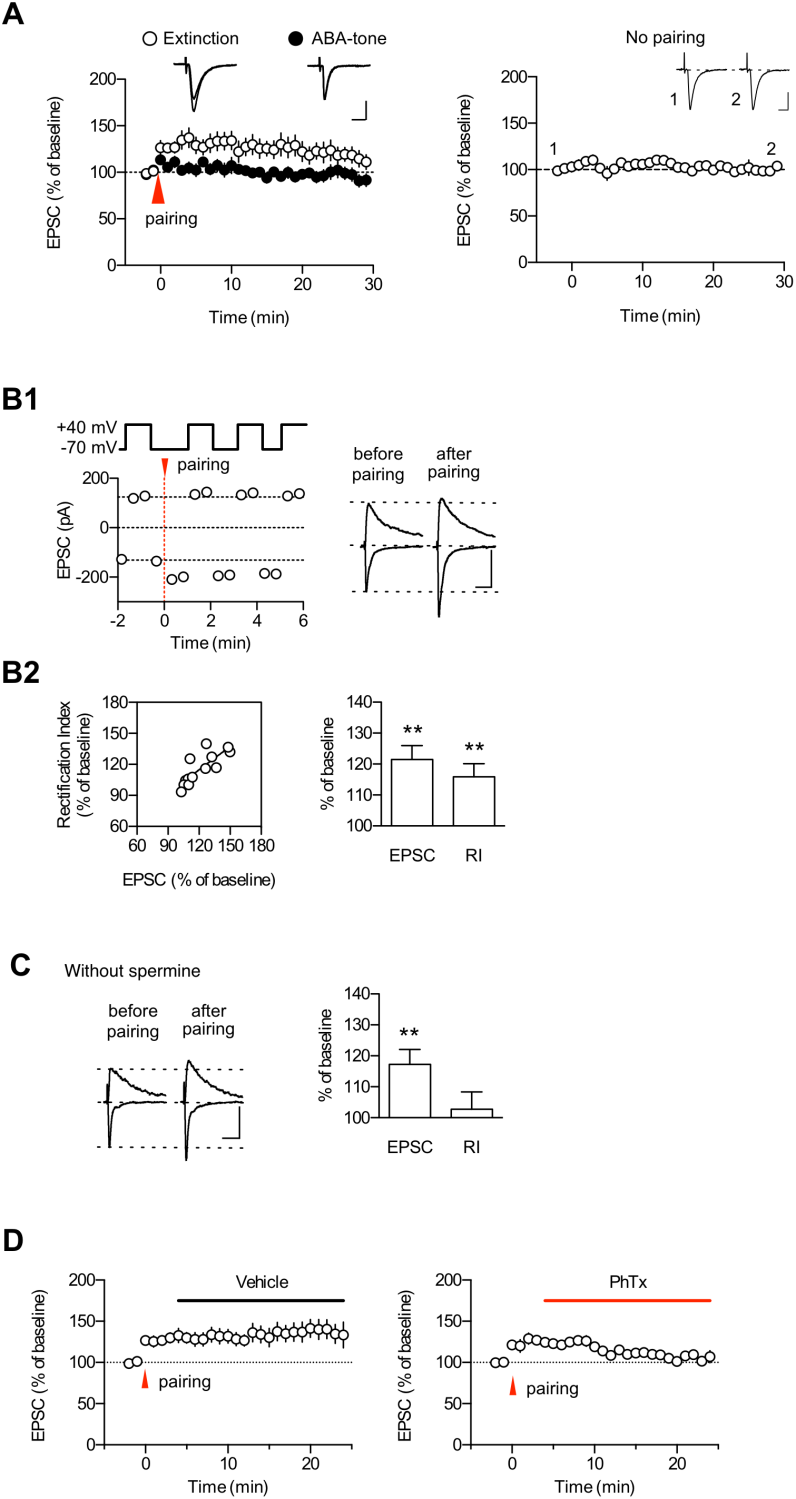
The GluR2-lacking AMPAR activity is also enhanced during low-threshold potentiation. **A.** The pairing protocol induced low-threshold potentiation, and ABA renewal occluded the low-threshold potentiation. Left, Pairing of four stimulus pulses (100 Hz) with a postsynaptic depolarization (40 ms) induced significantly less potentiation in the renewal group than in the extinction controls. The baseline responses for the first 3-min period were omitted because these responses tended to show a spontaneous increase after starting the whole-cell recordings. Instead, the baseline responses for 2 min before the delivery of the pairing protocol are shown (the pairing protocol was given 5 min after start of the whole-cell recordings). Right, The baseline responses were stable at least for 30 min in slices prepared from the extinction group. Representative paired traces are the averages of four traces within 2 min before and 25 min after pairing, respectively. Scale bars, 10 ms and 50 pA. **B.** The low-threshold potentiation was associated with an enhancement in the RI of EPSCs recorded at −70 mV and +40 mV (see Methods for additional details). **B1** Left: A representative experiment in which the RI was estimated during low-threshold potentiation using an internal solution with spermine. **B1** Right: Superimposed averages of EPSCs for a neuron shown in the left graph. **B2** Left: The magnitude of the low-threshold potentiation and the rectification index were significantly correlated (r2 = 0.6473). **B2** Right: Summary data (n = 13) for the RI changes after the induction of low-threshold potentiation. **C.** Left, Summary data (n = 12) for the RI changes after the induction of low-threshold potentiation in the experiments without intracellular spermine. Right, Example traces. In this experiment, we observed a significant increase (>130% of baseline responses) in NMDA EPSCs after pairing in 25% of cells recorded and ruled out these cells from further analyses. In **B** and **C**, D-AP5 was not included in the recording solution. *, p<0.05; **, p<0.01 (paired t-test). Scale bars in **B** and **C**, 50 ms and 100 pA. **D.** PhTx inhibited enhanced EPSCs after the induction of the low-threshold potentiation. EPSCs were elicited at a frequency of 0.33 Hz.

In our previous study on ABC renewal [29], we have proposed and characterized low-threshold potentiation as a cellular substrate for fear renewal. The logics behind this are as follows. Renewal is provoked by much weaker stimuli than those needed for fear memory formation. One possible mechanism for renewal is a metaplastic process [35] in which previous learning stimuli (extinction of conditioned fear) to LA synapses produce a long-term decrease in the threshold for the subsequent induction of plasticity for ABA renewal. Thus, the induction threshold for synaptic potentiation would be lowered in slices prepared after extinction training. The pairing protocol consisted of a 40-ms postsynaptic depolarization paired with four stimulus pulses at 100 Hz and delivered 5 min after the start of each whole-cell recoding (see Materials and Methods). The pairing protocol produced a low-threshold potentiation in slices prepared from the extinction group (1 d after the last extinction session), consistent with our previous work [29]. We also reasoned that if renewal involves and uses up low-threshold potentiation mechanisms, it is possible that ABA renewal occludes the low-threshold potentiation. The same pairing protocol produced significantly less potentiation in slices prepared from the renewal group (which was performed 1 d after the last extinction session) than from the extinction group (first 10 min, extinction group, 130.5±7.8% of baseline, n = 9; ABA-tone group, 106.4±4.2% of baseline, n = 10; p<0.02, unpaired t-test; last 10 min, extinction group, 119.0±8.8% of baseline; ABA-tone group, 97.0±5.7% of baseline; p<0.05, unpaired t-test; Figure 4A left). The EPSC amplitudes were not altered when the recordings were performed under the same conditions as the extinction group in Figure 4A (left) except that the pairing protocol was not applied (100.4±4.87%, n = 7, p = 0.9435, paired t-test; Figure 4A right). In sum, our data suggest that the extinction of conditioned fear renders a threshold of T-LA synaptic potentiation lowered considerably and further indicate that the low-threshold potentiation may underlie ABA renewal.

To determine whether the same RI changes as those observed with ABA renewal occur after the induction of low-threshold potentiation, we monitored changes in the RI before and after the induction of the low-threshold potentiation. The low-threshold potentiation produced a significant potentiation of EPSCs recorded at −70 mV, but a much smaller change in EPSCs was recorded at +40 mV (Figure 4B1). In addition, the low-threshold potentiation did not cause a significant change in the NMDAR-mediated synaptic currents measured 100 ms after the start of EPSCs recorded at +40 mV (107.6±4.8% of baseline, p = 0.13787, paired t-test). Using the ratio of peak EPSC amplitudes at −70 mV and +40 mV as a measure of the RI, we found enhancements in the RI after the induction of the low-threshold potentiation (115.9±4.2% of baseline, p = 0.00271, paired t-test; Figure 4B2); these findings suggest enhancements in GluA2-lacking AMPAR activity at the LA synapses after the induction of low-threshold potentiation. The detection of the inward rectification of GluR2-lacking AMPARs depends on the inclusion of spermine in the intracellular solution because endogenous polyamines are rapidly dialyzed from neurons during whole-cell recordings. To ensure that the change in the RI is due to a polyamine block of GluR2-lacking AMPARs, we performed a control experiment in which the intracellular solution did not contain spermine (Figure 4C). In this condition, no consistent changes were observed in either the RI (102.8±5.6% of baseline, p = 0.6275, paired t-test) or the NMDAR-meditated EPSCs (107.1±4.1% of baseline, p = 0.1125, paired t-test) after low-threshold potentiation (Figure 4C).

We also examined the effects of PhTx after the induction of low-threshold potentiation in the extinction group. The application of PhTx beginning 5 min after pairing inhibited the EPSC amplitude (p = 0.0058, paired t-test; Figure 4D right). The control experiments showed a stable expression of low-threshold potentiation (p = 0.2763, paired t-test; Figure 4D left). Taken together, these findings, along with the lack of PhTx effects on basal transmission in slices prepared from the extinction group (Figure 3B), suggest that GluA2-lacking AMPAR activity is enhanced after the induction of low-threshold potentiation.

### Blockade of ABA Renewal via the Inhibition of GluA2-lacking AMPAR Activity

**Figure 5:**
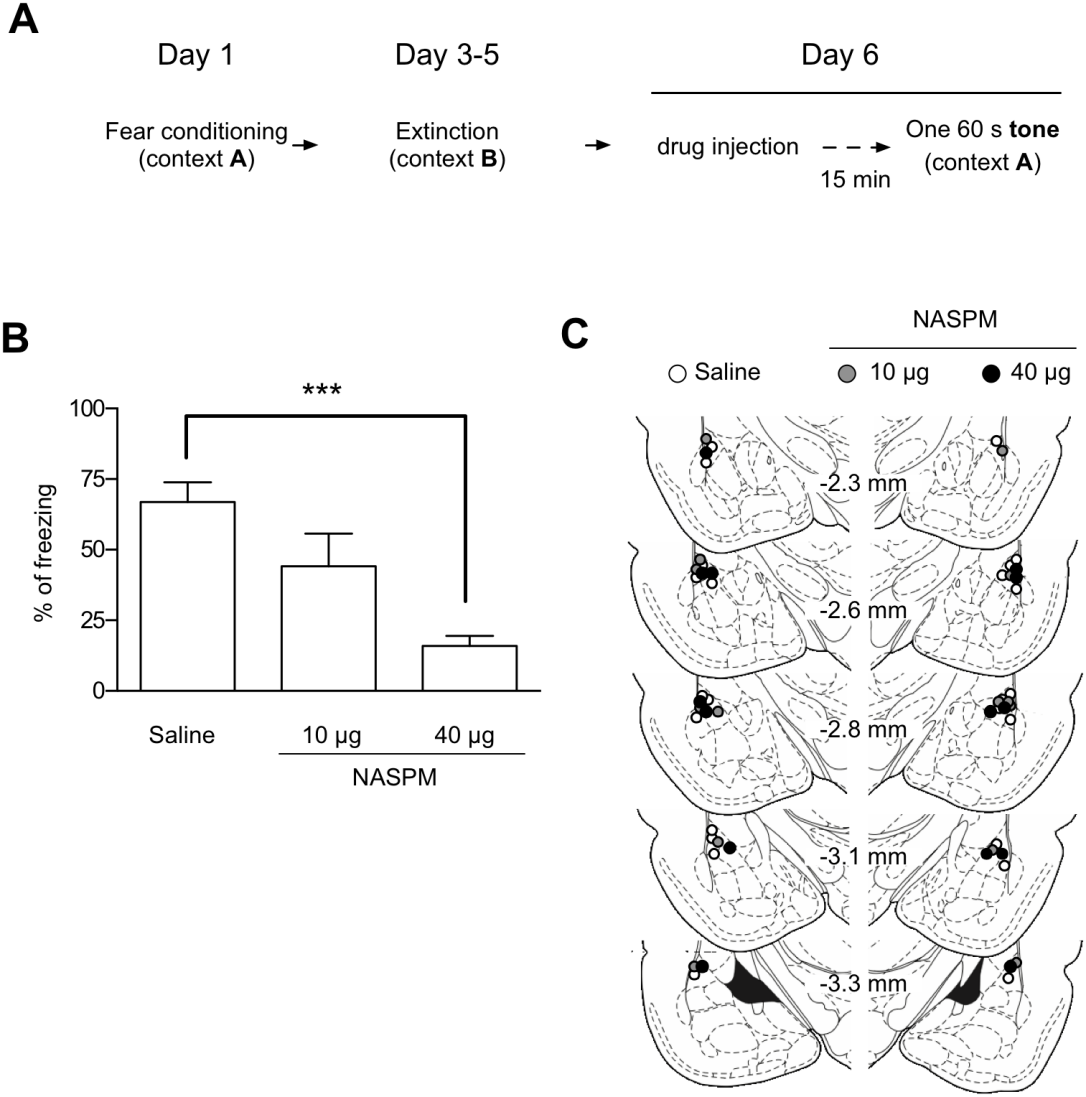
Inhibition of GluR2-lacking AMPAR activity in the LA is required for ABA renewal. **A.** The behavioral procedure for the experiments. NASPM, a selective blocker of GluA2-lacking AMPARs, was microinjected 15 min before tone presentation in context C on Day 6. In this figure, a weaker conditioning protocol was used (see Methods for additional details). **B.** Microinjection of NASPM (40 µg) into the LA impaired ABA renewal relative to the microinjection of saline (F_2,23_ = 10.44, p = 0.0006, one-way ANOVA; Saline, 66.9±7.0%, n = 12; 10 µg NASPM, 44.1±11.6%, n = 7; 40 µg NASPM, 16.0±3.5%, n = 7; p<0.001, Newman-Keuls post-test). **C.** Schematic representation of the injector cannula tips. Histological plates illustrating the injection site in the LA were adopted from the rat brain atlas [50].

We next sought to determine whether enhancements in GluA2-lacking AMPAR activity are required for ABA renewal. To test this hypothesis, we microinjected either NASPM or vehicle into the LA 15 min before the tone presentation in the ABA renewal protocol (Figure 5A). The concentration of NASPM for microinjection was chosen based on a previous report [36]. The bilateral microinjection of NASPM (40 µg/0.5 µl/each side) into the LA inhibited ABA renewal relative to vehicle controls, while a lower concentration of NASPM (10 µg/0.5 µl/each side) failed to do so (Figure 5B, C). These results suggest that the activity of GluA2-lacking AMPARs is required for ABA renewal.

### Critical Role of GluA1 Phosphorylation at Ser831 in both Low-threshold Potentiation and ABA Renewal

**Figure 6:**
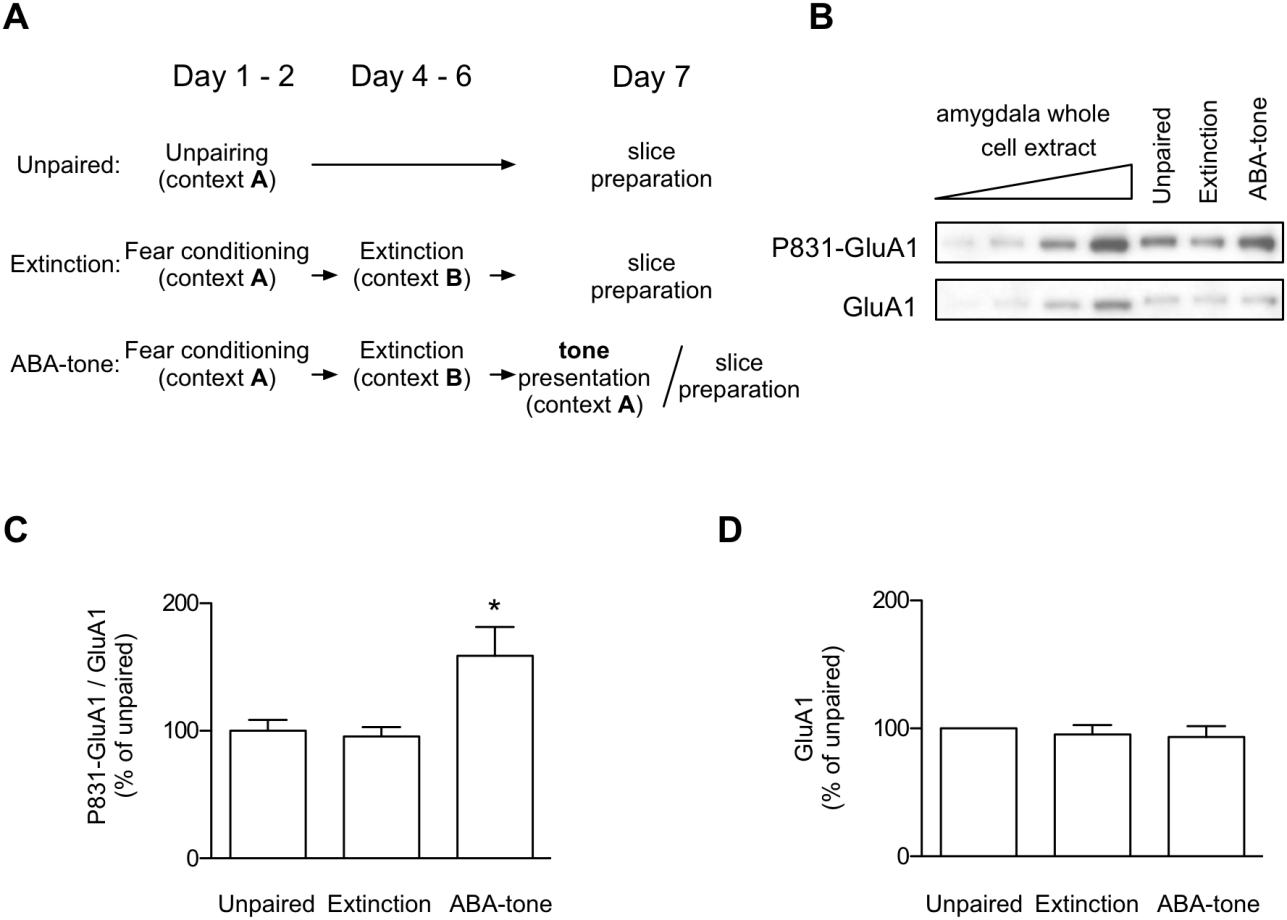
ABA renewal-inducing stimuli enhance Ser-831 phosphorylation of surface GluR1 in the LA synaptosomes. **A.** The behavioral procedure. On Day 7, the LA synaptosomal membranes were prepared immediately after a tone test (ABA-tone group). In the unpaired and extinction groups, one set of rats was killed for the preparation of LA synaptosomal membranes, while another set was monitored for conditioned freezing to CS on Day 7. **B.** Representative immunoblot. **C.** Pooled results showing that Ser-831 phosphorylation was enhanced upon ABA renewal. *, p<0.05, one-way ANOVA (F_2,12_ = 5.850, p = 0.0169) followed by Newman-Keuls post-test. **D.** There was no significant difference across the three groups in terms of the amount of surface GluR1 from the LA synaptosomes. The number of rats used in each group was as follows: unpaired = 20, extinction = 18, renewal = 19.

Next, we investigated whether the Ser831 phosphorylation of GluA1 would be enhanced upon ABA renewal, similar to the case of ABC renewal [29]. To monitor changes in the phosphorylation of the synaptic surface AMPARs upon renewal, we isolated the surface proteins of the LA synaptosomes using a biochemical surface biotinylation technique [25], [29], [37], [38] in three groups: unpaired control, extinction and ABA-tone groups (Figure 6A). Ser831 phosphorylation of the synaptic surface GluA1 was significantly increased in the renewal group relative to the unpaired or extinction groups (F_2,12_ = 5.850, p = 0.0169, one-way ANOVA; p<0.05 for the ABA tone group vs. other group, p>0.05 for the unpaired group vs. extinction group, Newman-Keuls post-test) (Figure 6B, C), without any changes in the expression of synaptic surface GluA1 (Figure 6D). Thus, ABA renewal appears to be associated with enhancements in Ser-831 phosphorylation.

To determine whether Ser831-phosphorylation is required for either renewal or its cellular substrate, we employed a GluA1-derived peptide, GluA1_S_ (LIPQQ**S** ^831^INEAI). We have reasoned that because GluA1_S_ mimics the Ser831-included C-tail region of GluA1 subunit, the serine residue in GluA1_S_ can also be phosphorylated upon renewal; thus, the phosphorylated GluA1_S_ peptide may compete with Ser831-phosphorylated GluA1 to prevent the proper interaction between Ser831-phosphorylated GluA1 and its adaptor molecules (see [29], [39]–[41]). As a non-competitive control, we used GluA1_A_ (LIPQQ**A** ^831^INEAI), in which the serine was replaced with an alanine residue.

**Figure 7:**
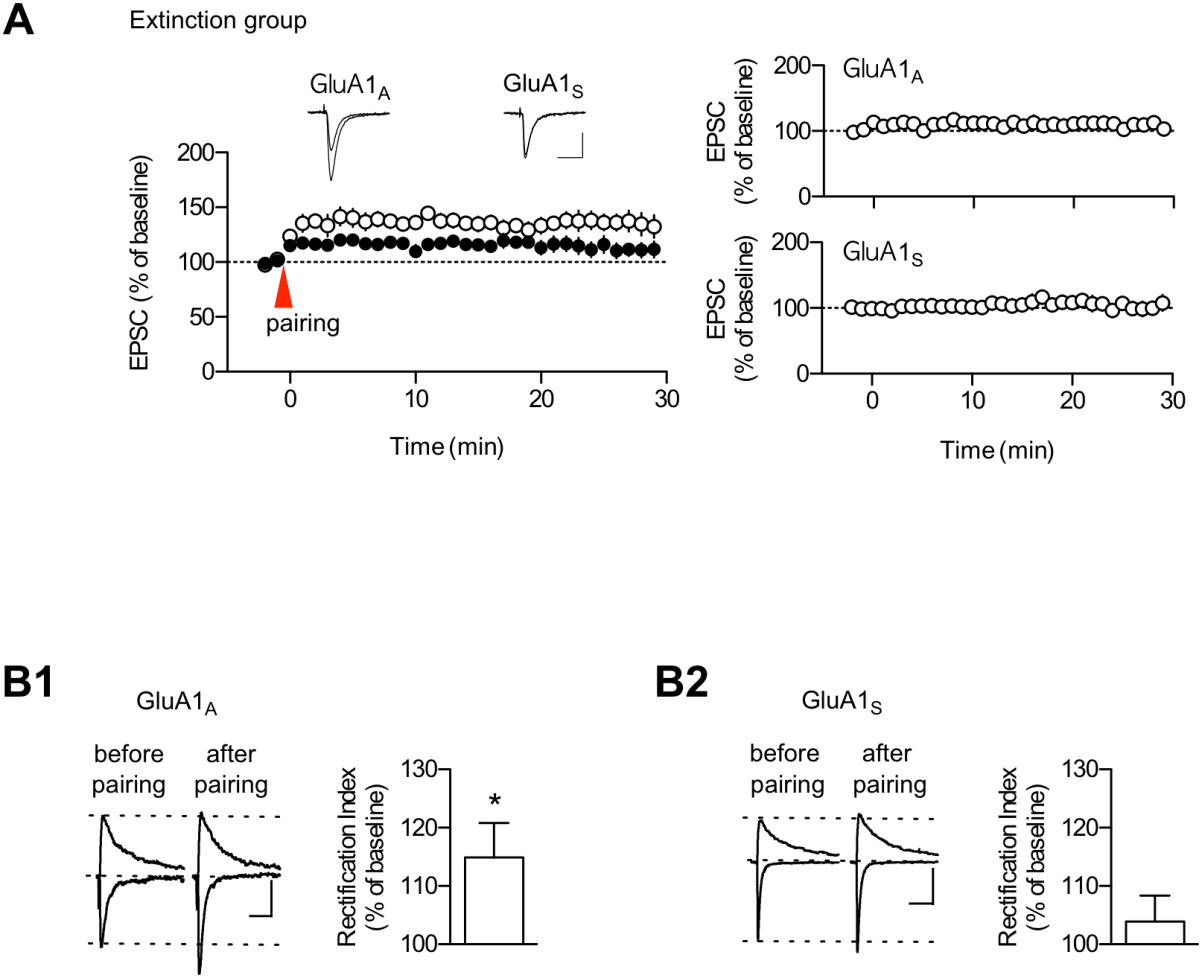
The GluR1_S_ peptide inhibits both low-threshold potentiation and its associated enhancement in the RI. **A.** Left: Inclusion of GluA1_S_ in the internal solution inhibited the low-threshold potentiation relative to GluA1_A_ (GluR1_A_, 135.9±7.8% of baseline, n = 12; GluA1_S_, 113.3±6.6% of baseline, n = 16). Representative traces are superimposed averages of the EPSCs before and 20 min after pairing. Scale bars, 20 ms and 50 pA. Right: The inclusion of GluA1_S_ or GluA1_A_ in the internal solution did not have a significant effect on basal synaptic transmission (GluA1s, 104.1±6.7% of baseline, n = 5, p>0.5; GluA1_A_, 109.7±5.8 of baseline, n = 5, p>0.1; paired *t*-test). **B1**. The pairing protocol produced an enhancement in the RI and the AMPA EPSC amplitudes when cells were dialyzed with GluA1_A_. Left: Sample traces from an individual experiment. Right: Summary data for the RI changes after the induction of low-threshold potentiation (114.9±5.9% of baseline, n = 13). NMDAR-meditated EPSCs did not change significantly after the induction of low-threshold potentiation (113.4±7.4% of baseline, p = 0.0930, paired *t*-test). **B2**. The inclusion of GluA1_S_ in the internal solution attenuated the RI increase associated with the low-threshold potentiation. Left, Sample traces from an individual experiment. Right, Summary data for the RI changes after the induction of low-threshold potentiation (103.9±4.5% of baseline, n = 13). NMDAR-meditated EPSCs did not change (106.9±4.6% of baseline, p = 0.1563, paired *t*-test). *, p<0.05.

We then used this peptide to determine whether enhancements in Ser831 phosphorylation would be required for low-threshold potentiation. The inclusion of GluA1_S_ peptide (300 µg/ml) in the internal solution also blocked the low-threshold potentiation (Figure 7A) compared with GluA1_A_ (300 µg/ml) (p<0.05, unpaired t-test), while both peptides had no significant effect on basal transmission in slices prepared from extinguished rats. This finding indicates that low-threshold potentiation may require enhanced Ser831 phosphorylation. We then tested the possibility that the enhancement in the GluA2-lacking AMPAR activity during low-threshold potentiation (see Figure 4) is also attenuated by the inclusion of the GluA1_S_ peptide. As predicted, while a significant increase in the RI in the presence of GluA1_A_ was observed during low-threshold potentiation, the RI increase during low-threshold potentiation was blocked by the inclusion of GluA1s peptide in the internal solution, but not by the inclusion of GluA1_A_ (Figure 7B1, B2). Together, these findings suggest that enhancements in Ser831 phosphorylation are required for both the low-threshold potentiation and enhanced GluA2-lacking AMPAR activity.

**Figure 8:**
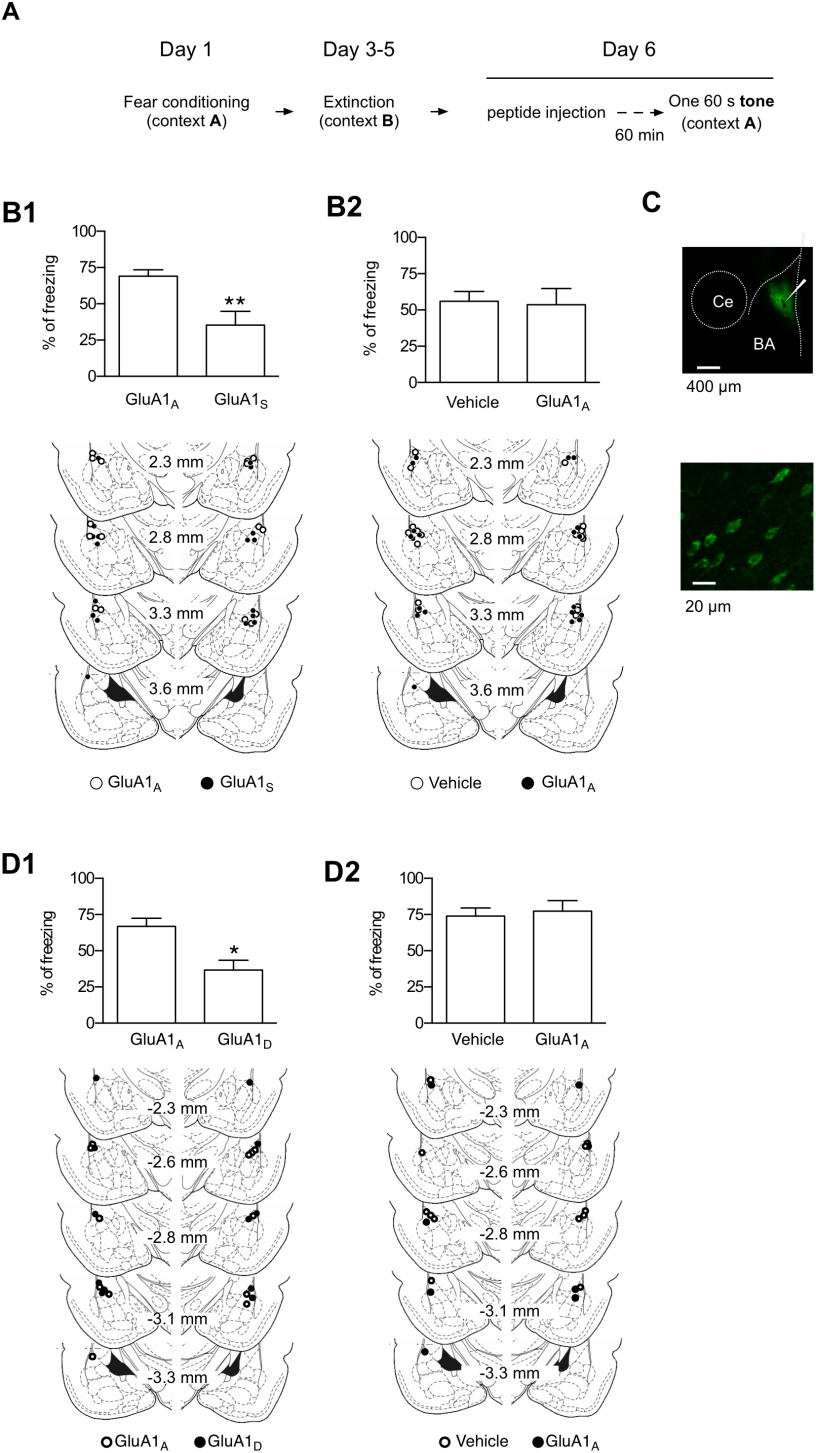
The microinjection of a cell permeable form of the GluA1-derived peptides into the LA attenuates ABA renewal. **A.** The behavioral procedure. The tone stimulus used for renewal was 60****sec in duration on Day 6. In this figure, a weaker conditioning protocol was used (*see* Methods for additional details). **B1**. Upper, The microinjection of GluA1_S_ into the LA impaired ABA renewal relative to GluA1_A_ (GluA1_A_, 41.43±2.68%, n = 8; GluA1_S_, 21.24±5.63%, n = 9; p<0.01, unpaired *t*-test). Lower, Schematic representation of the injector cannula tips. Histological plates illustrating the injection site in the LA were adopted from the rat brain atlas [50] (○, GluA1_A_; •, GluA1_S_). **B2**. Top, GluA1_A_ peptide injection had no effects on ABA renewal relative to vehicle controls (vehicle, 56.09±6.78%, n = 9; GluA1_A_, 53.70±11.05%, n = 10; p>0.5, unpaired *t*-test). Bottom, Schematic representation of the injector cannula tips. Histological plates illustrating the injection site in the LA were adopted from the rat brain atlas (○, Vehicle; •, GluA1_A_). **C.** Diffusion of the fluorescent dansyl-tat-GluR1_S_ peptide (1****nmol) within 1****h after the microinjection, as visualized with a multiphoton microscope (top). The white arrow indicates the end of the injector cannula. Peptide transduction in individual LA neurons at high magnification (bottom). Ce, central amygdala; BA, basal amygdala. **D1**. Top: Microinjection of GluA1_D_ into the LA impaired ABA renewal relative to GluA1_A_ (GluA1_A_, 66.82±5.62%, n = 6; GluA1_D_, 36.70±6.77%, n = 6; p<0.01, unpaired t-test). Bottom: Schematic representation of the injector cannula tips (○, GluA1_A_; •, GluA1_D_). **D2**. Top: GluA1_A_ injection had no effects on ABA renewal relative to vehicle controls (vehicle, 74.01±5.65%, n = 6; GluA1_A_, 77.37±7.24%, n = 4; p>0.05, unpaired t-test). Bottom: Schematic representation of the injector cannula tips (○, Vehicle; •, GluA1_A_).

Finally, we reasoned that if enhancements in Ser831 phosphorylation are required for ABA renewal, then ABA renewal would be attenuated by the GluA1_S_ peptide. To test this hypothesis, we performed an intracranial microinfusion of a cell-permeable form of GluA1_S_ or GluA1_A_ (Tat-GluA1_S_ or Tat-GluA1_A_; 15 pmol, 60 min before the tone presentation for renewal; Figure 8A) into the LA. Microinfusion of GluA1_S_ into the LA attenuated ABA renewal compared with the control peptide (GluA1_A_)-infused group (p<0.01, unpaired t-test; Figure 8B1), although the contextual freezing level measured during the acclimation period was not significantly different between the two groups (p>0.05, unpaired t-test). To determine whether the control peptide itself has any effects on ABA renewal, we microinjected GluA1_A_ (or vehicle) into the LA and did not find any significant effect of the peptide on renewal compared with the vehicle controls (p>0.5, unpaired t-test; Figure 8B2). Diffusion of the injected peptide was analyzed using fluorescent dansyl-Tat-GluA1_S_ peptide with a multiphoton microscope (Figure 8C).

In addition, we tested another GluA1-derived peptide, GluA1_D_ (LIPQQ**D** ^831^INEAI), which had been shown to attenuate ABC renewal when microinjected into the LA [29]. In the case of GluA1_D_, the serine is replaced with an aspartate residue, a phosphomimetic amino acid, and this peptide appears to compete with Ser831-phosphorylated GluA1 [29]. The microinfusion of GluA1_D_ into the LA attenuated ABA renewal compared with the control peptide (GluA1_A_)-infused group (p<0.01, unpaired t-test; Figure 8D1), although the contextual freezing level measured during the acclimation period was not significantly different between the two groups (p>0.05, unpaired t-test). To determine whether the control peptide itself has any effects on ABA renewal, we microinjected GluA1_A_ (or vehicle) into the LA and did not find any significant effect of the peptide on renewal compared with vehicle controls (p>0.5, unpaired t-test; Figure 8D2). Together, these findings suggest that the enhancements in the Ser-831 phosphorylation of GluA1 in the LA are required for ABA renewal.

## Discussion

Our findings suggest that similar mechanisms underlie both ABA renewal and ABC renewal; that is, enhancements in both the GluA2-lacking AMPAR activity and GluA1 phosphorylation at Ser831 (see also [29]). In this study, we have made several new findings concerning the molecular mechanisms underlying renewal. Most importantly, we have found that GluA2-lacking AMPAR activity is required for renewal, as evidenced by the attenuation of ABA renewal through the microinjection of NASPM into the LA. We have also elaborated the mechanisms of low-threshold potentiation, a proposed cellular substrate for renewal. Low-threshold potentiation is accompanied by enhancements in GluA2-lacking AMPAR activity; furthermore, low-threshold potentiation is maintained mainly by enhanced GluA2-lacking AMPAR activity because PhTx treatment after the induction of low-threshold potentiation blocked most of the potentiated responses (see Figure 4).

Unique biophysical properties of GluR2-lacking AMPARs (e.g., high single-channel conductance and high calcium permeability) have important implications for both the physiology and pathology of fear renewal. It has been predicted that adding GluR2-lacking AMPARs numbering <5% of existing synaptic AMPARs is sufficient to account for a 80% potentiation of synaptic transmission [42], indicating that GluR2-lacking AMPARs are efficient and potent in altering synaptic efficacy. This receptor is therefore an optimal cellular substrate for engendering rapid and strong behavioral responses such as fear renewal. Calcium permeability of this receptor is thought to be important for both synaptic plasticity and neuropathology. The enhanced activity of GluR2-lacking AMPARs upon renewal may contribute to the regional brain atrophies observed in some patients with post-traumatic stress disorder [43]. GluA2-lacking AMPARs have also been implicated in a number of different physiological and pathological processes in vivo, such as memory consolidation [44] and reconsolidation [45], cocaine addiction [36], and ischemic cell death [46].

We have proposed that the GluA1_S_ peptide exerts its effect via its phosphorylation at Ser831 upon renewal and that the phosphorylated GluA1_S_ peptide competes with endogenous Ser831-phosphorylated GluA1. Although we cannot rule out other possible mechanisms underlying the inhibitory actions of GluA1_S_ on renewal, the GluA1_D_ experiment (see Figure 8D) supports our conclusion that Ser831 phosphorylation is required for ABA renewal, given that the GluA1_D_ peptide has been shown to specifically attenuate ABC renewal, possibly by hindering the interaction between Ser831-phosphorylated GluA1 and its adaptors (see [29], [39]–[41]).

In sum, our present and previous findings indicate that the LA plays a critical role in fear renewal. As a molecular mechanism, we propose that enhancements in both GluA2-lacking AMPAR activity and GluA1 phosphorylation at Ser831 at the LA synapses are responsible for eliciting fear renewal and that this particular molecular mechanism appears to be preserved in different induction protocols to evoke fear renewal.

## Materials and Methods

### Behavioral Procedures

All procedures were approved by the Institute of Laboratory Animal Resources of Seoul National University (Korea). Male Sprague-Dawley rats (4–5 weeks old) were maintained with free access to food and water under an inverted 12/12 hr light/dark cycle (lights off at 09∶00 hrs). Behavioral training was done during the dark portion of the light/dark cycle. For fear conditioning in all experiments except those shown in Figure 5 and 8, rats were placed in a conditioning chamber and left undisturbed for 2 min. Then, a neutral tone (30 s, 2.8 kHz, 85 dB) coterminating with an electrical foot shock (1.0 mA, 1 s) was presented three times at an average interval of 100 s (Day 1). The three tone-shock pairings were repeated the next day (Day 2). At each of the days, rats were returned to their home cages 60 s after the last shock was applied. For extinction training (Days 4 through 6), rats were placed in the new chamber, allowed to settle for 4 min, and then presented with 20 (Day 4) and 15 shock-free tones (Day 5 and 6), respectively, at an average interval of 100 s. A Plexiglas chamber distinct from conditioning chamber was used for both extinction training and tone test. For renewal (Day 7), extinguished rats were placed back in the conditioning chamber for 10 min, and then given the same neutral tone for 30 s, but without a foot shock. Brain slices were prepared immediately after a tone test (ABB-tone and ABA-tone groups) or context exposure (ABC-context group) on Day 7. In the remaining groups (unpaired controls and extinction groups), one set of rats was killed for the preparation of brain slices, and another set was used for monitoring of conditioned freezing, which was taken as evidence of a fear response. Conditioned freezing was defined as immobility except for respiratory movements, and was quantified by trained observers who were blind to the experimental groups. Total freezing time during a test period was normalized versus the duration of the tone presentation (30 s) or context exposure. For the experiments shown in Figure 5 and 8, 10 week-old rats were fear-conditioned using weaker parameters (1.0 mA and 0.5 s) and the first day-scheduled conditioning was skipped (i.e., these rats only received three tone-shock pairings on the second day in the behavioral protocol shown in Figure 1A), and a tone stimulus for renewal was 60 s in duration.

### Cannula Implantation and Peptide Infusion

Rats were anesthetized with an intraperitoneal (i.p.) injection of pentobarbital sodium (50 mg/kg) and mounted on a stereotaxic apparatus (Stoelting, Wood Dale, IL, USA); 26-gauge stainless-steel cannulas (model C315G; Plastics One Products, Roanoke, VA, USA) were implanted bilaterally into the LA (AP: −3.0 mm, ML: ±5.15 mm and DV: −7.0 mm) using previously described techniques [25], [47]. A 32-gauge dummy cannula was inserted into each cannula to prevent clogging. Two jewelry screws were implanted over the skull to serve as anchors, and the whole assembly was affixed on the skull with dental cement. Rats were given at least one week to recover before the experiments were performed. Following the completion of the experiments, the intra-LA placement of the injection cannula tips was confirmed. Briefly, rats were anesthetized with urethane (1 g/kg, i.p.) and transcardially perfused with 0.9% saline solution followed by 10% buffered formalin. Brains were removed and post-fixed overnight. Coronal sections (80-µm-thick) were cut using a vibroslicer (NVSL, World Precision Instruments, Sarasota, FL, USA), stained with cresyl violet, and examined under a light microscope. For the microinfusion of peptides, Tat-GluA1-derived peptides (Peptron, Daejeon, South Korea) were dissolved in artificial cerebrospinal fluid (aCSF). The peptides were administered bilaterally into the LA via 33 gauge injector cannulas (C315I; Plastic Products), which was attached to a 10 µl Hamilton syringe, at a rate of 0.25 µl/min (60 min before the tone presentation in the renewal group or the start of the open field test). Following peptide infusion, the cannulas were left in place for an additional minute to allow the peptides to diffuse away from the cannula tip. The dummy cannulas were then replaced, and the rats were returned to their home cages.

### Slice Preparation

Brain slices were prepared using previously described techniques [29]. In brief, Sprague-Dawley rats (4–5 weeks old) were anesthetized with isoflurane and decapitated. Whole brains were isolated and placed in an ice-cold modified aCSF solution containing (in mM) 175 sucrose, 20 NaCl, 3.5 KCl, 1.25 NaH_2_PO_4_, 26 NaHCO_3_, 1.3 MgCl_2,_ 11 D-(+)-glucose, and was gassed with 95% O_2_/5% CO_2_. Coronal slices (300 µm) including the LA were cut using a vibroslicer (HA752, Campden Instruments, Loughborough, UK) and incubated in normal aCSF containing (in mM) 120 NaCl, 3.5 KCl, 1.25 NaH_2_PO_4_, 26 NaHCO_3_, 1.3 MgCl_2_, 2 CaCl_2_, 11 D-(+)-glucose, and was continuously bubbled at room temperature with 95% O_2_/5% CO_2_. Just before a given slice was transferred to the recording chamber, the cortex overlying the LA was cut away with a scalpel, so the addition of picrotoxin (100 µM; Sigma-Aldrich, St. Louis, MO, USA) would block cortical epileptic burst discharges from invading the LA.

### Afferent Stimulation and Recording Conditions

We chose brain slices containing a well-isolated, sharply defined trunk (containing thalamic afferents) crossing the dorsolateral division of the LA where the somatosensory and auditory inputs converge [48]. The sizes of the LA and the central amygdala were relatively constant in the utilized slices; when multiple trunks were observed, we used the closest trunk to the central nucleus of the amygdala. Unless otherwise noted, the thalamic afferents were stimulated at a frequency of 0.067 Hz using a concentric bipolar electrode (MCE-100, Rhodes Medical Instruments, Summerland, CA, USA). The stimulation electrode was placed at the midpoint of the trunk between the internal capsule and the medial boundary of the LA. The regions and cells of interest for all recordings were located beneath the midpoint of the trunk spanning the LA horizontally.

### Whole-cell Patch-clamp Recordings

Whole-cell recordings were made using an Axopatch 200A amplifier or Multiclamp 700A (Molecular Devices, Sunnyvale, CA, USA). For experiments that required a higher quality of voltage clamping (i.e., the experiments in Figures 1D, 2B, 3), the recordings were obtained using a Cs-based internal solution containing (in mM) 100 Cs-gluconate, 0.6 EGTA, 10 HEPES, 5 NaCl, 20 TEA, 4 Mg-ATP, 0.3 Na-GTP and 3 QX314, with the pH adjusted to 7.2 with CsOH and the osmolarity adjusted to approximately 297 mmol/kg with sucrose. In the remaining experiments, the pipettes were filled with a K-based internal solution containing (in mM) 120 K-gluconate, 0.2 EGTA, 10 HEPES, 5 NaCl, 1 MgCl_2_, 2 Mg-ATP and 0.3 Na-GTP, with the pH adjusted to 7.2 with KOH. The cells used were classified as principal neurons based on the pyramidal shape of their somata, their ability to show spike-frequency adaptation in response to current injection in potassium-filled cells and the slower decay time of spontaneous EPSCs in cesium-filled cells (see also supplementary texts in [25] for additional details). We included picrotoxin (100 µM) in our recording solution to isolate excitatory synaptic transmission and block feed-forward GABAergic inputs to the principal neurons in the LA. The pipette resistances ranged from 2.5 to 3.5 M ohm. IR-DIC-enhanced visual guidance was used to select neurons that were three to four cell layers below the surface of the 300-µm-thick slices, which were held at 32±1°C. The neurons were voltage-clamped at –70 mV, and the various solutions were delivered to the slices via gravity-driven superfusion at a flow rate of 1.5 ml/min. The pipette series resistance was monitored throughout each experiment, and the data were discarded if it changed by >20%. Whole-cell currents were filtered at 1 kHz, digitized at up to 20 kHz, and stored on a microcomputer (Clampex 8 software, Molecular Devices, Sunnyvale, CA, USA). Pairing-induced low-threshold potentiation was induced by four individual stimulus pulses at 100 Hz with postsynaptic depolarization (0 or −10 mV for 40 ms). One or two neurons were recorded per animal (a single neuron per slice). The pairing protocol was delivered 5 min after the start of each whole-cell recording; the pairing protocol failed to induce low-threshold potentiation when applied >5 min after the start of the whole-cell recording, possibly due to a “washout” effect (data not shown; see also ref. [49]). During the first 3 min after the start of the whole-cell recording, the amplitude of the baseline responses was set to approximately 150 pA. Data points collected from 3 to 5 min after the start of the recording were used as a baseline, and recordings that showed a baseline drift of >10% were discarded. All recordings were completed within 3.5 hrs after slice preparation, mainly due to the cell viability of the 300-µm-thick slices. For better display, the running averages of four or six data points were applied in the time-lapse experiments.

### Analysis of Evoked mEPSCs

AMPAR-mediated, asynchronous evoked mEPSCs were collected during a 400-ms period beginning 50 ms after each stimulus, which consisted of a 1.67-Hz, 10-pulse train delivered once every 30 s in a bath solution containing D-AP5 (50 µM, Tocris, Ellisville, MO, USA), 5 mM MgCl_2_ and 3 mM Sr^2+^ (or D-AP5, 5 mM MgCl_2_ and 3 mM Ca^2+^ for the measurement of synchronous evoked EPSCs prior to each Sr^2+^ experiment). Quantal events were analyzed using Minianalysis software (Synaptosoft, Decatur, GA, USA) with the detection parameters set at an amplitude >6 pA and a rise time <3 ms; the results were visually verified. For each cell, a random stretch of 300 mEPSCs was used to construct a cumulative probability plot and to calculate the mean mEPSC amplitude. Data from the cumulative EPSC histograms were statistically compared using the Kolmogorov-Smirnov test.

### Estimation of the RI

The rectification index (RI) was calculated as the ratio of the peak amplitudes (EPSC_hyperpolarized_/EPSC_depolarized_) obtained with an internal solution that contained spermine (100 µM). The reversal potential (E_rev_) was measured in each experiment. The RI (E_rev_ −60 mV/E_rev_+40 mV) was compared 20 min after the start of the whole-cell recordings to ensure the complete diffusion of the exogenous spermine into the cell interior. D-AP5 (100 µM) was applied 5 min before the RI estimation, which allowed us to isolate the AMPAR-mediated EPSCs at positive potentials.

### Biochemical Measurements of Surface AMPA Receptors on the LA Synaptosomal Membranes

Biotinylation experiments monitoring the expression of surface AMPA receptors were performed as described previously [29], [37], [38] with modifications. LA areas microdissected from 400-µm-thick brain slices were pooled (three to four pieces per animal), incubated with aCSF containing 1 mg/ml sulfosuccinimidyl-6-(biotinamido) hexanoate (Pierce Chemical Company, Rockford, IL, USA) for 30 min on ice, and then quenched by two successive 20-min washes in aCSF containing 100 mM glycine, followed by two washes in ice-cold TBS (50 mM Tris, pH 7.5, and 150 mM NaCl). The microdissected LAs were then lysed in ice-cold homogenization buffer containing (in mM) 10 Tris (pH 7.6), 320 sucrose, 5 NaF, 1 Na_3_VO_4_, 1 EDTA, and 1 EGTA. A 10 µg aliquot of each lysate was retained as a total protein fraction, and the remainder was centrifuged at 1000×*g* for 10 min at 4°C for the removal of nuclei and large debris. The supernatant was further centrifuged at 10,000×*g* at 4°C for 30 min to obtain a crude synaptosomal fraction, which was then lysed in modified RIPA buffer containing (in mM) 50 Tris (pH 7.6), 150 NaCl, 5 NaF, 1 Na_3_VO_4_, 0.5% Triton X-100, 0.5% sodium deoxycholate, 0.1% SDS, 1 phenylmethylsulfonyl fluoride, 100 µg/ml aprotinin, and 100 µg/ml leupeptin. The samples were sonicated and spun down at 15,000×*g* at 4°C for 15 min. The supernatant was mixed with 400 µl of modified RIPA buffer and 100 µl of 50% Neutravidin agarose (Pierce Chemical Company); it was then incubated for 3 hrs at 4°C to isolate the biotinylated proteins from the crude synaptosomal complexes. The Neutravidin agarose was washed four times with modified RIPA buffer, and the bound proteins were eluted with SDS sample buffer by boiling for 5 min. The isolated biotinylated proteins were subsequently analyzed by immunoblotting with monoclonal anti-GluA1 (1∶1000; Santa Cruz Biotechnology, Santa Cruz, CA, USA) and polyclonal anti-p-Ser-831 (1∶1000; Millipore, Billerica, MA, USA). The immunoblot was probed with a HRP-conjugated secondary antibody (Jackson Immunoresearch Laboratories, Inc., PA, USA) for 1 hr and developed using an ECL-based immunoblotting detection system (Pierce Chemical Company). The relative optical densities of the bands were quantified using the ImageJ image analysis software (National Institutes of Health, Bethesda, MD, USA). We confirmed the equal loading of proteins based on the densitometric quantification of silver-stained band profiles obtained from gels that were pre-run with small aliquots of the loaded samples. The linearity of the immunoblotting results was confirmed by analyzing the relative optical band densities of serially diluted samples (amygdala whole-cell extracts) loaded on each gel. The optical densities of the GluA1 bands in the extinction and renewal groups were normalized with respect to those of the unpaired group in each experiment.

### Statistical Analysis

Between-group comparisons of data were made using either an unpaired t-test or one-way ANOVA with subsequent Newman-Keuls post-hoc comparison. A paired t-test was used to determine whether the post-treatment responses differed significantly from the baseline responses. A p-value <0.05 was considered statistically significant. The data from each neuron/slice were treated as independent samples. In all experiments with behaviorally trained rats, the data included samples from three or more animals.

## References

[1] Hermans D, Craske MG, Mineka S, Lovibond PF (2006) Extinction in human fear conditioning. Biol Psychiatry 60: 361–368 10.1016/j.biopsych.2005.10.006 16503330

[2] Vervliet B, Craske MG, Hermans D (2013) Fear extinction and relapse: state of the art. Annu Rev Clin Psychol 9: 215–248 10.1146/annurev-clinpsy-050212-185542 23537484

[3] Bouton ME (2004) Context and behavioral processes in extinction. Learn Mem 11: 485–494 10.1101/lm.78804 15466298

[4] Corcoran KA, Maren S (2001) Hippocampal inactivation disrupts contextual retrieval of fear memory after extinction. J Neurosci 21: 1720–1726. 11222661 10.1523/JNEUROSCI.21-05-01720.2001 PMC6762930

[5] Herry C, Ciocchi S, Senn V, Demmou L, Müller C, et al (2008) Switching on and off fear by distinct neuronal circuits. Nature 454: 600–606 10.1038/nature07166 18615015

[6] Knapska E, Maren S (2009) Reciprocal patterns of c-Fos expression in the medial prefrontal cortex and amygdala after extinction and renewal of conditioned fear. Learn Mem 16: 486–493 10.1101/lm.1463909 19633138 PMC2726014

[7] Cole S, Richardson R, McNally GP (2013) Ventral hippocampal kappa opioid receptors mediate the renewal of fear following extinction in the rat. PLoS ONE 8: e58701 10.1371/journal.pone.0058701 23675405 PMC3651202

[8] Cole S, Richardson R, McNally GP (2011) Kappa opioid receptors mediate where fear is expressed following extinction training. Learn Mem 18: 88–95 10.1101/lm.2049511 21245209

[9] Harris JA, Jones ML, Bailey GK, Westbrook RF (2000) Contextual control over conditioned responding in an extinction paradigm. J Exp Psychol Anim Behav Process 26: 174–185. 10782432 10.1037//0097-7403.26.2.174

[10] Thomas BL, Larsen N, Ayres JJB (2003) Role of context similarity in ABA, ABC, and AAB renewal paradigms: Implications for theories of renewal and for treating human phobias* 1. Learning and Motivation 34: 410–436 Available: http://www.sciencedirect.com/science/article/pii/S0023969003000377.

[11] Fonteyne R, Baeyens F (2011) Dissociations between ABA-, ABC-, and AAB-renewal of Pavlovian modulation in human sequential feature positive discrimination learning. Exp Psychol 58: 278–286 10.1027/1618-3169/a000094 21310693

[12] Havermans RC, Keuker J, Lataster T, Jansen A (2005) Contextual control of extinguished conditioned performance in humans. Learning and Motivation 36: 1–19 10.1016/j.lmot.2004.09.002

[13] Neumann DL (2006) The effects of physical context changes and multiple extinction contexts on two forms of renewal in a conditioned suppression task with humans. Learning and Motivation 37: 149–175 10.1016/j.lmot.2005.06.004

[14] Üngör M, Lachnit H (2008) Dissociations among ABA, ABC, and AAB recovery effects. Learning and Motivation.

[15] Wilson A, Brooks DC, Bouton ME (1995) The role of the rat hippocampal system in several effects of context in extinction. Behav Neurosci 109: 828–836. 8554708 10.1037//0735-7044.109.5.828

[16] Frohardt RJ, Guarraci FA, Bouton ME (2000) The effects of neurotoxic hippocampal lesions on two effects of context after fear extinction. Behav Neurosci 114: 227–240 10.1037//0735-7044.114.2.227 10832785

[17] Corcoran KA, Maren S (2004) Factors regulating the effects of hippocampal inactivation on renewal of conditional fear after extinction. Learn Mem 11: 598–603 10.1101/lm.78704 15466314 PMC523078

[18] Ehlers A, Hackmann A, Steil R, Clohessy S, Wenninger K, et al (2002) The nature of intrusive memories after trauma: the warning signal hypothesis. Behav Res Ther 40: 995–1002. 12296496 10.1016/s0005-7967(01)00077-8

[19] Ledoux JE (2000) Emotion circuits in the brain. Annu Rev Neurosci 23: 155–184 10.1146/annurev.neuro.23.1.155 10845062

[20] Nader K, Majidishad P, Amorapanth P, Ledoux JE (2001) Damage to the lateral and central, but not other, amygdaloid nuclei prevents the acquisition of auditory fear conditioning. Learn Mem 8: 156–163 10.1101/lm.38101 11390635 PMC311372

[21] Wilensky AE, Schafe GE, Ledoux JE (1999) Functional inactivation of the amygdala before but not after auditory fear conditioning prevents memory formation. J Neurosci 19: RC48. 10594092 10.1523/JNEUROSCI.19-24-j0006.1999 PMC6784952

[22] Rogan MT, Stäubli UV, Ledoux JE (1997) Fear conditioning induces associative long-term potentiation in the amygdala. Nature 390: 604–607 10.1038/37601 9403688

[23] McKernan MG, Shinnick-Gallagher P (1997) Fear conditioning induces a lasting potentiation of synaptic currents in vitro. Nature 390: 607–611 10.1038/37605 9403689

[24] Mao S-C, Chang C-H, Wu C-C, Orejanera MJ, Manzoni OJ, et al (2013) Inhibition of Spontaneous Recovery of Fear by mGluR5 after Prolonged Extinction Training. PLoS ONE 8: e59580 10.1371/journal.pone.0059580.g007 23555716 PMC3605338

[25] Kim J, Lee S, Park K, Hong I, Song B, et al (2007) Amygdala depotentiation and fear extinction. Proc Natl Acad Sci USA 104: 20955–20960 10.1073/pnas.0710548105 18165656 PMC2409248

[26] Maren S, Quirk GJ (2004) Neuronal signalling of fear memory. Nat Rev Neurosci 5: 844–852 10.1038/nrn1535 15496862

[27] Myers KM, Davis M (2006) Mechanisms of fear extinction. Mol Psychiatry 12: 120–150. 17160066 10.1038/sj.mp.4001939

[28] Sotres-Bayon F, Sierra-Mercado D, Pardilla-Delgado E, Quirk GJ (2012) Gating of fear in prelimbic cortex by hippocampal and amygdala inputs. Neuron 76: 804–812 10.1016/j.neuron.2012.09.028 23177964 PMC3508462

[29] Lee S, Song B, Kim J, Park K, Hong I, et al (2013) GluA1 phosphorylation at serine 831 in the lateral amygdala is required for fear renewal. Nat Neurosci 16: 1436–1444 10.1038/nn.3491 23974710

[30] Mahanty NK, Sah P (1998) Calcium-permeable AMPA receptors mediate long-term potentiation in interneurons in the amygdala. Nature 394: 683–687 10.1038/29312 9716132

[31] Kamboj SK, Swanson GT, Cull-Candy SG (1995) Intracellular spermine confers rectification on rat calcium-permeable AMPA and kainate receptors. J Physiol (Lond) 486 (Pt 2): 297–303. 7473197 10.1113/jphysiol.1995.sp020812 PMC1156521

[32] Bowie D, Mayer ML (1995) Inward rectification of both AMPA and kainate subtype glutamate receptors generated by polyamine-mediated ion channel block. Neuron 15: 453–462 10.1016/0896-6273(95)90049-7 7646897

[33] Koh DS, Burnashev N, Jonas P (1995) Block of native Ca (2+)-permeable AMPA receptors in rat brain by intracellular polyamines generates double rectification. J Physiol (Lond) 486 (Pt 2): 305–312. 7473198 10.1113/jphysiol.1995.sp020813 PMC1156522

[34] Tóth K, McBain CJ (1998) Afferent-specific innervation of two distinct AMPA receptor subtypes on single hippocampal interneurons. Nat Neurosci 1: 572–578 10.1038/2807 10196564

[35] Abraham WC, Bear MF (1996) Metaplasticity: the plasticity of synaptic plasticity. Trends Neurosci 19: 126–130. 8658594 10.1016/s0166-2236(96)80018-x

[36] Conrad K, Tseng K, Uejima J, Reimers J, Heng L, et al (2008) Formation of accumbens GluR2-lacking AMPA receptors mediates incubation of cocaine craving. Nature. 10.1038/nature06995 PMC2574981 18500330

[37] Chung HJ, Xia J, Scannevin RH, Zhang X, Huganir RL (2000) Phosphorylation of the AMPA receptor subunit GluR2 differentially regulates its interaction with PDZ domain-containing proteins. J Neurosci 20: 7258–7267. 11007883 10.1523/JNEUROSCI.20-19-07258.2000 PMC6772789

[38] Dunah AW, Standaert DG (2001) Dopamine D1 receptor-dependent trafficking of striatal NMDA glutamate receptors to the postsynaptic membrane. J Neurosci 21: 5546–5558. 11466426 10.1523/JNEUROSCI.21-15-05546.2001 PMC6762635

[39] Kristensen AS, Jenkins MA, Banke TG, Schousboe A, Makino Y, et al (2011) Mechanism of Ca2+/calmodulin-dependent kinase II regulation of AMPA receptor gating. Nat Neurosci 14: 727–735 10.1038/nn.2804 21516102 PMC3102786

[40] Derkach VA, Oh MC, Guire ES, Soderling TR (2007) Regulatory mechanisms of AMPA receptors in synaptic plasticity. Nat Rev Neurosci 8: 101–113 10.1038/nrn2055 17237803

[41] Kessels HW, Kopec CD, Klein ME, Malinow R (2009) Roles of stargazin and phosphorylation in the control of AMPA receptor subcellular distribution. Nat Neurosci 12: 888–896 10.1038/nn.2340 19543281 PMC3922706

[42] Guire ES, Oh MC, Soderling TR, Derkach VA (2008) Recruitment of calcium-permeable AMPA receptors during synaptic potentiation is regulated by CaM-kinase I. J Neurosci. 28: 6000–6009 10.1523/JNEUROSCI.0384-08.2008 PMC2671029 18524905

[43] Sapolsky RM (2001) Atrophy of the hippocampus in posttraumatic stress disorder: how and when? Hippocampus 11: 90–91 10.1002/hipo.1026 11345129

[44] Clem RL, Huganir RL (2010) Calcium-permeable AMPA receptor dynamics mediate fear memory erasure. Science 330: 1108–1112 10.1126/science.1195298 21030604 PMC3001394

[45] Hong I, Kim J, Kim J, Lee S, Ko H-G, et al (2013) AMPA receptor exchange underlies transient memory destabilization on retrieval. Proc Natl Acad Sci USA 110: 8218–8223 10.1073/pnas.1305235110 23630279 PMC3657785

[46] Noh K-M, Yokota H, Mashiko T, Castillo PE, Zukin RS, et al (2005) Blockade of calcium-permeable AMPA receptors protects hippocampal neurons against global ischemia-induced death. Proc Natl Acad Sci USA 102: 12230–12235 10.1073/pnas.0505408102 16093311 PMC1189338

[47] Kim J, Lee S, Park H, Song B, Hong I, et al (2007) Blockade of amygdala metabotropic glutamate receptor subtype 1 impairs fear extinction. Biochem Biophys Res Commun 355: 188–193 10.1016/j.bbrc.2007.01.125 17292864

[48] Pitkänen A, Savander V, Ledoux JE (1997) Organization of intra-amygdaloid circuitries in the rat: an emerging framework for understanding functions of the amygdala. Trends Neurosci 20: 517–523. 9364666 10.1016/s0166-2236(97)01125-9

[49] Malinow R, Tsien RW (1990) Presynaptic enhancement shown by whole-cell recordings of long-term potentiation in hippocampal slices. Nature 346: 177–180 10.1038/346177a0 2164158

[50] Paxinos G, Watson C (1997) The Rat Brain in Stereotaxic Coordinates. San Diego: Academic Press. 1 p.

